# Frequency domain analysis of steady-state visual evoked potentials in dogs with optic neuritis: a pilot study

**DOI:** 10.3389/fvets.2025.1603620

**Published:** 2025-06-24

**Authors:** Teck-Geun Lee, Joon-Young Kim

**Affiliations:** Department of Veterinary Ophthalmology, College of Veterinary Medicine, Konkuk University, Seoul, Republic of Korea

**Keywords:** optic neuritis, steady-state visual evoked potentials, transient visual evoked potentials, signal-to-noise ratio, response amplitude

## Abstract

Visual evoked potentials (VEP) are electrophysiological signals used to assess visual pathway function, with applications in diagnosing optic nerve disorders. This study compared the diagnostic utility of transient VEP (TVEP) and steady-state VEP (SSVEP) in dogs with optic neuritis, focusing on SSVEP’s frequency-domain advantages. Seven dogs with optic neuritis and seven controls matched for breed, age, and weight were evaluated. TVEP and SSVEP were recorded without anesthesia using standardized protocols, and parameters were analyzed in time and frequency domains. Significant reductions in SSVEP Frequency-Domain amplitude (FD amplitude), Time-Domain amplitude (TD amplitude), and Signal-to-Noise Ratio (SNR) were observed in the optic neuritis group (FD amplitude: *p* < 0.001, TD amplitude: *p* < 0.001, SNR: *p* < 0.001). TVEP N1-P2 amplitude was also significantly lower in the optic neuritis group (*p* < 0.001), while P2 latency showed no significant differences. Indirect comparisons revealed that TVEP amplitudes were larger, likely due to noise artifacts in time-domain analysis. SSVEP demonstrated superior noise resistance and minimized subjective interpretation. These results suggest SSVEP’s potential as a reliable, non-invasive diagnostic tool for optic neuritis in dogs, with further studies needed to confirm its broader applications in veterinary ophthalmology.

## 1 Introduction

Visual evoked potentials (VEP) are electrophysiological signals generated in the visual cortex in response to visual stimulation. They assess the function of the visual pathway from the retina to the visual cortex and are particularly useful for diagnosing optic nerve disorders (1).

VEP is classified into transient VEP (TVEP) and steady-state VEP (SSVEP) based on the frequency of the visual stimulus (2). TVEP, elicited at frequencies below 3 Hz, produces distinct neural responses to each stimulus (2). In dogs, it typically appears as an M-shaped waveform with five peaks, which vary in latency and amplitude depending on factors such as age or pathology (3, 4). In contrast, SSVEP is elicited at frequencies above 3 Hz, where a new response is generated before the previous one has fully resolved. This overlap in responses creates a continuous, sinusoidal waveform (2).

One of the key distinctions between TVEP and SSVEP is their method of analysis (5, 6). TVEP is evaluated in the time domain, focusing on latency and amplitude. However, this approach can be subjective, especially when waveforms are complex or noisy, making both recording and interpretation more difficult (7). A recent study also reported that TVEP is subject to considerable inter-individual variability, which may limit its clinical applicability (8). These limitations may contribute to the limited clinical use of VEP in veterinary practice, despite its sensitivity to optic neuropathy (9).

In contrast, SSVEP uses frequency-domain analysis to isolate response amplitudes and reduce background noise. This reduces interpretive subjectivity and enhances signal stability. Because of these advantages, SSVEP has become a widely used tool for assessing visual perception and ophthalmic disorders in humans (8, 10–12). These characteristics suggest that SSVEP may address some of the limitations associated with conventional VEP and serve as a useful tool for the diagnosis of optic neuropathy in veterinary medicine.

A representative example is optic neuritis, an inflammatory disease of the optic nerve that causes sudden vision impairment in dogs, often accompanied by reduced pupillary light reflex (PLR) and funduscopic changes, such as optic nerve head swelling, retinal edema, and retinal hemorrhages (13). While it can be associated with conditions such as meningoencephalitis of unknown etiology (MUE) or immune-mediated inflammation, its precise cause is frequently unclear (13). Diagnosis in veterinary ophthalmology typically involves comprehensive ophthalmic examinations, including funduscopic assessments, magnetic resonance imaging (MRI), and cerebrospinal fluid analysis, to differentiate optic neuritis from other optic nerve or retinal diseases (14).

This study aimed to evaluate the diagnostic utility of SSVEP in dogs with optic neuritis, comparing it to TVEP. We assessed group differences in the respective parameters of SSVEP and TVEP. Additionally, we compared the time-domain and frequency-domain parameters of SSVEP with those of TVEP to evaluate their potential as diagnostic tools for optic neuritis in veterinary ophthalmology.

## 2 Materials and methods

### 2.1 Animals

The medical records of optic neuritis cases evaluated with VEP at the Veterinary Medical Teaching Hospital (VMTH), Konkuk University, from December 2020 to April 2025 were reviewed for inclusion in this study. As a routine procedure, the hospital requested the owners of all animals enrolled in the study to fill out a patient consent form, which included a clause that patient information obtained during treatment may be used for research purposes. Information was collected only from owners who submitted the completed consent form. This study was conducted in accordance with all applicable regulations and guidelines, and all animals were treated in compliance with the ARVO Statement for the Use of Animals in Ophthalmic and Vision Research. All procedures were approved by the Institutional Animal Care and Use Committee of Konkuk University (approval no. KU23018).

Dogs in the optic neuritis group were diagnosed based on comprehensive ophthalmic examinations, including the Schirmer tear test I, applanation tonometry, fluorescein dye test, slit-lamp biomicroscopy, direct and indirect ophthalmoscopy, and electro-retinography (ERG). ERG was performed without sedation or anesthesia to assess gross retinal function, following the ECVO protocol (15). Only dogs that met our laboratory’s criteria for normal retinal function were included in the study. Optical coherence tomography (OCT) was performed in some cases to evaluate retinal layers and thickness. Additionally, MRI was used to confirm optic nerve involvement and assess the extent of optic neuritis.

This study included seven dogs diagnosed with optic neuritis, confirmed by ophthalmic findings, clinical signs, and MRI, which provided additional evidence of potential optic nerve involvement. One dog was diagnosed with MUE, with concurrent optic neuritis and brain lesions. Visual deficits in this case were primarily attributed to optic nerve dysfunction, based on ophthalmic findings and localized MRI abnormalities.

Dogs were excluded if their visual deficits were solely due to cerebral lesions or non-inflammatory optic nerve disorders. For example, two dogs with brain edema and hydrocephalus secondary to traumatic brain injury were excluded, as MRI findings indicated these conditions as the primary cause of visual impairment. Additionally, dogs with optic nerve tumors or optic nerve atrophy were excluded, as these conditions did not meet the criteria for optic neuritis.

All tested eyes in the optic neuritis group exhibited severe visual impairment, with 13 out of 14 eyes showing absent menace response and dazzle reflex. PLR was absent in all but one eye, which showed a reduced PLR with a partial dazzle reflex, highlighting significant visual deficits.

The control group consisted of dogs with conditions that did not impair vision, such as mild blepharitis, epiphora, and nasal fold trichiasis. All control dogs had normal vision, as confirmed by neurological and ophthalmic examinations, with no evidence of diseases causing visual impairment. Of the seven dogs in the control group, two were receiving topical preventives and five were receiving oral preventives as part of routine parasite control. None of the dogs were receiving corticosteroids (either systemic or topical), immunosuppressants, or other medications that could affect visual function at the time of examination. Control dogs were matched with the optic neuritis group based on breed, age, and body weight.

### 2.2 VEP recording

TVEP and SSVEP were recorded without sedation or anesthesia using the RETI-port-Scan 21 (Roland Consult, Brandenburg, Germany), following the method described by Strain (16). All dogs were fasted for at least 8 h prior to hospital admission according to the standard protocol of our institution. Following topical instillation of 0.5% tropicamide and 0.5% phenylephrine (Mydrin-P, Santen Pharmaceutical Co., Ltd., Osaka, Japan), the dogs rested in the resting area under ambient lighting until full pupil dilation was confirmed. VEP recordings were initiated after confirmation of adequate mydriasis. Gentle handling techniques were employed throughout the recording procedures to minimize stress and promote behavioral stability. Three needle electrodes were used for VEP recordings: the active electrode was placed on the occipital scalp over the visual cortex (Oz), the reference electrode on the frontal region (Fpz), and the ground electrode on the vertex (Cz). The dogs were maintained in a stable position by manual restraint, with the handler supporting the mandible and the back of the neck. To minimize potential artifacts, care was taken to ensure that neither the handler’s nor the examiner’s hands came into contact with the area surrounding the electrodes. Electrode impedance was kept below 5 kΩ during recording. The recordings were conducted in a dark room, and monocular stimulation was applied to the right eye using a handheld stimulator (MINI Ganzfeld I8, Roland Consult, Brandenburg, Germany), while the left eye was covered to prevent light exposure. Flash strength was set to 3 cd·s/m^2^ according to ISCEV standards, with stimulation frequencies of 1.6 Hz for TVEP and 8 Hz for SSVEP.

TVEP was recorded first in the right eye, followed by SSVEP. After completing the right eye measurements, the same procedures were repeated for the left eye, with the right eye covered. A minimum of 50 responses was averaged for each recording. The signals were amplified, bandpass-filtered (1–100 Hz), and analyzed using the RETIport/scan 21 software (Roland Consult, Berlin, Germany). The software automatically identified three positive and two negative peaks in the TVEP recordings. When automatic peak identification was unsuccessful, a single examiner manually determined the peaks with reference to waveform morphology and the latency and amplitude ranges described by Strain et al. and Kimotsuki et al., which have been widely used as normative standards in canine FVEP studies (3, 4, 16). The examiner was blinded to group assignments during the marking process. Artifact contamination was assessed visually without applying fixed amplitude criteria, and decisions were based primarily on the shape and polarity of positive and negative peaks.

### 2.3 Description of VEP parameters

SSVEP parameters were analyzed using both frequency-domain and time-domain methods. Frequency-domain parameters included frequency-domain amplitude (FD amplitude), signal-to-noise ratio (SNR), and phase latency, while the time-domain parameter was the time-domain amplitude (TD amplitude).

The FD amplitude, previously referred to as the response amplitude in prior SSVEP studies, represents the peak amplitude of the 1st harmonic at the stimulus frequency in the frequency-domain spectrum (10). This parameter is derived through Fourier analysis and quantifies cortical activity synchronized with the stimulus frequency, effectively isolating the neural response from unrelated noise components (10). All frequency-domain parameters were obtained from the fully automated outputs provided by the RETIport software, without applying any user-defined FFT settings, to ensure transparency and reproducibility.

The SNR quantifies the quality of the evoked response by comparing the response power at the stimulus frequency to the noise power in surrounding frequency bands. An SNR value of ≥ 3 is considered valid for reliable frequency-domain analysis, ensuring that the recorded responses are not noise-dominated (10, 17).

The phase latency reflects the temporal delay of neural activity relative to stimulus onset, measured in milliseconds (ms). Phase latency captures the time difference between the stimulus and the neural response, providing insights into the timing of cortical processes. This parameter is derived from spectral analysis and is commonly used to evaluate temporal synchronization in SSVEP studies. (10, 18).

The TD amplitude, representing the peak-to-peak difference in the time-domain SSVEP waveform, was used in this study to allow indirect comparisons with TVEP parameters. Previous studies on 10 Hz FVEPs have demonstrated that time-domain amplitude is a reliable predictor of visual function, even in cases of media opacity such as vitreous hemorrhage or cataracts (19, 20). Based on these findings, TD amplitude was selected as the primary time-domain parameter for comparing SSVEP and TVEP.

TVEP parameters were analyzed in the time domain. The primary parameters were N1–P2 amplitude, defined as the peak-to-peak difference between the N1 trough and the P2 peak, and P2 latency, defined as the time from stimulus onset to the P2 wave peak.

N1–P2 amplitude is widely used in TVEP studies to assess cortical response magnitude (3, 21), while P2 latency reflects the timing and conduction speed of neural transmission along the visual pathway (4, 21, 22). These two parameters were selected for group comparison, as previous studies have reported significant alterations in both amplitude and latency in cases of visual pathway impairment. Due to the transient nature of TVEP, frequency-domain analysis was not applicable, and the RETI-port system used in this study did not support such analysis.

### 2.4 Statistical analysis

All statistical analyses were performed using SPSS version 30 (IBM Corp., Armonk, NY, USA). Continuous variables were tested for normality using the Shapiro–Wilk test. Differences in general characteristics, including age, body weight, and sex, between the optic neuritis and control groups were analyzed using Fisher’s exact test for categorical variables and independent t-test or Mann–Whitney U test for continuous variables, depending on the normality of the data. Group differences in TVEP and SSVEP parameters were evaluated using Generalized Estimating Equations (GEE), which account for within-subject correlations and repeated measures. Pairwise comparisons were conducted between FD and TD amplitudes from SSVEP, and between TD amplitude from SSVEP and N1–P2 amplitude from TVEP, using the Wilcoxon signed-rank test due to the non-parametric distribution of the data. To account for the increased likelihood of Type I errors due to multiple comparisons, Bonferroni correction was applied. Accordingly, the adjusted significance threshold was set at *p* < 0.025 for comparisons between FD and TD amplitude, as well as TD and N1-P2 amplitude. A *p*-value of < 0.05 was considered statistically significant for all other analyses.

## 3 Results

### 3.1 Demographic and clinical characteristics

A total of 14 dogs (28 eyes) were included in the study, with 7 dogs (14 eyes) in the optic neuritis group and 7 dogs (14 eyes) in the healthy control group. The control group consisted of 5 females and 2 males, including Pomeranian (*n* = 2), Long-haired Chihuahua (*n* = 1), Maltese (*n* = 3), and Poodle (*n* = 1). The optic neuritis group included 4 females and 3 males, comprising Pomeranian (*n* = 3), Maltese (*n* = 3), and Poodle-Maltese mix (*n* = 1).

The mean age of the control group was 6.91 ± 2.46 years, while the optic neuritis group had a mean age of 7.31 ± 1.67 years. The average weights of the dogs were 3.57 ± 0.57 kg and 3.94 ± 1.27 kg (mean ± SD) in the control and optic neuritis groups, respectively. Statistical analysis revealed no significant differences in age (*p* = 0.729) or weight (*p* = 0.751) between the two groups.

### 3.2 SSVEP and TVEP parameters

**Figure 1:**
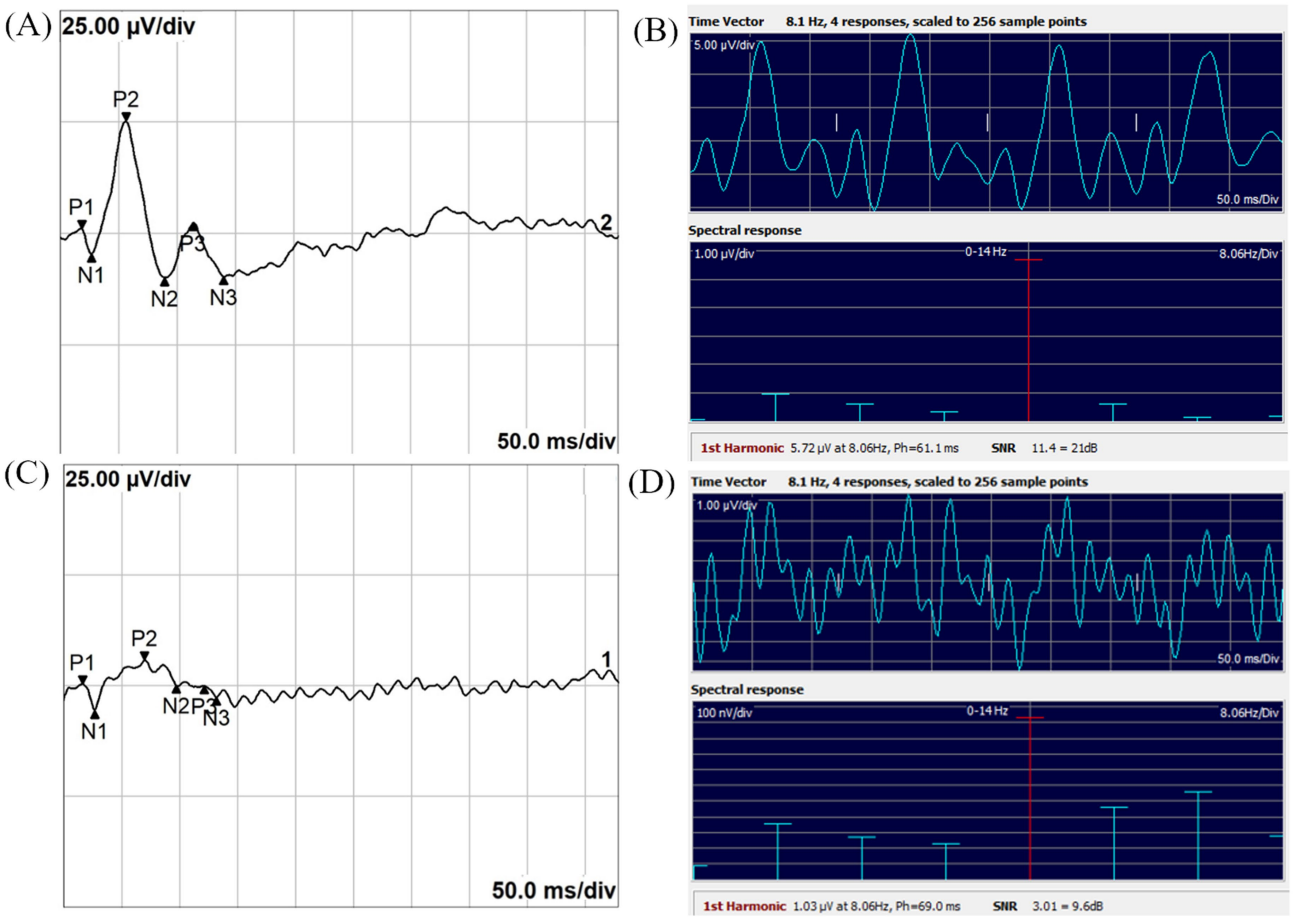
Representative images of TVEP and SSVEP in the Control and Optic Neuritis groups. **(A)** TVEP waveform recorded from the Control group. **(B)** SSVEP time-domain waveform (top panel) and frequency-domain spectral response (bottom panel) recorded from the Control group. **(C)** TVEP waveform recorded from the Optic Neuritis group. **(D)** SSVEP time-domain waveform (top panel) and frequency-domain spectral response (bottom panel) recorded from the Optic Neuritis group. The 1st harmonic, referred to as FD amplitude in this study, represents the amplitude at the stimulus frequency. Ph indicates the phase latency of the SSVEP, measured in milliseconds. SNR is the signal-to-noise ratio, provided both as a linear value and in decibels (dB).

Representative examples of TVEP and SSVEP waveforms in both control and optic neuritis groups are shown in Figure 1. These examples illustrate the visual differences in waveform clarity and spectral features between groups.

For SSVEP, the control group showed significantly higher FD amplitude and SNR compared to the optic neuritis group (RA: *p* < 0.001; SNR: *p* < 0.001), while the phase latency did not differ significantly (*p* = 0.244). The TD amplitude, derived from time-domain parameter, was also significantly greater in the control group (*p* < 0.001). Table 1 summarizes these findings.

**Table 1:** Comparison of SSVEP and TVEP Parameters between Control and Optic Neuritis Groups.

Parameter		Control	Optic neuritis	*p*-value
SSVEP	FD (μV)	5.07 ± 3.05	1.19 ± 0.26	< 0.001 ***
	SNR	10.71 ± 4.92	5.20 ± 3.50	< 0.001 ***
	Phase (ms)	62.15 ± 21.46	72.87 ± 16.48	0.244
	TD (μV)	20.17 ± 6.65	6.33 ± 3.08	< 0.001 ***
TVEP	N1-P2 (μV)	28.38 ± 6.20	9.20 ± 5.24	< 0.001 ***
	P2 latency (ms)	57.05 ± 12.31	55.56 ± 15.61	0.728

SSVEP, Steady-State Visual Evoked Potential; TVEP, Transient Visual Evoked Potential; FD, Frequency-Domain Amplitude; SNR, Signal-to-Noise Ratio; Phase, Phase Latency in SSVEP; TD, Time-Domain Amplitude in SSVEP; N1-P2, N1-P2 amplitude in TVEP. Values are expressed as mean ± standard deviation (SD). Statistical significance: *p < 0.05, **p < 0.01, ***p < 0.001.

For TVEP, the N1-P2 amplitude was significantly higher in the control group than in the optic neuritis group (*p* < 0.001), but P2 latency showed no significant difference between the groups (*p* = 0.728). Detailed comparisons are also presented in Table 1.

**Figure 2:**
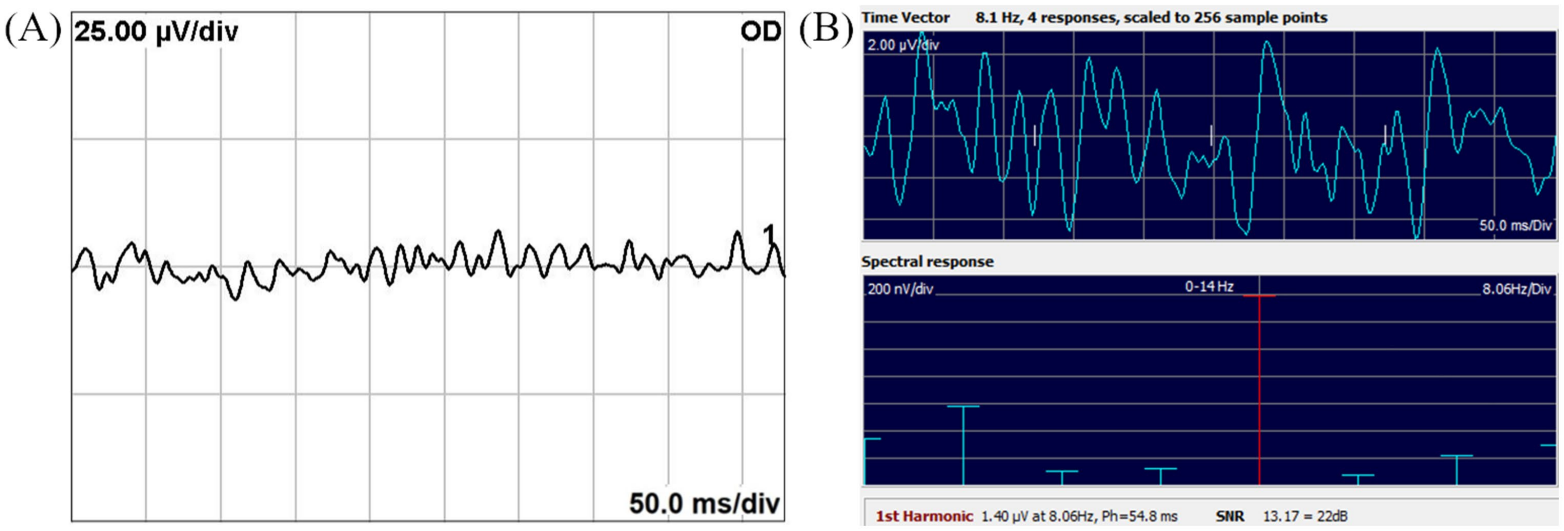
Noisy TVEP waveform **(A)** and corresponding SSVEP spectral response **(B)** from a dog with optic neuritis. **(A)** The TVEP waveform exhibits significant noise, hindering reliable peak identification and analysis. **(B)** The SSVEP frequency-domain spectral response demonstrates an SNR exceeding 3, highlighting its robustness in providing reliable neural activity measurements under noisy conditions. The 1st harmonic, referred to as FD amplitude in this study, represents the amplitude at the stimulus frequency. Ph indicates the phase latency of the SSVEP, measured in milliseconds. SNR is the signal-to-noise ratio, presented as both a linear value and in decibels (dB).

Figure 2 shows a noisy TVEP waveform from a dog with optic neuritis, where peak differentiation was difficult. The corresponding SSVEP measurements demonstrated an SNR exceeding 3, indicating reliable neural activity detection.

### 3.3 Amplitude comparisons within SSVEP and between SSVEP and TVEP

**Figure 3:**
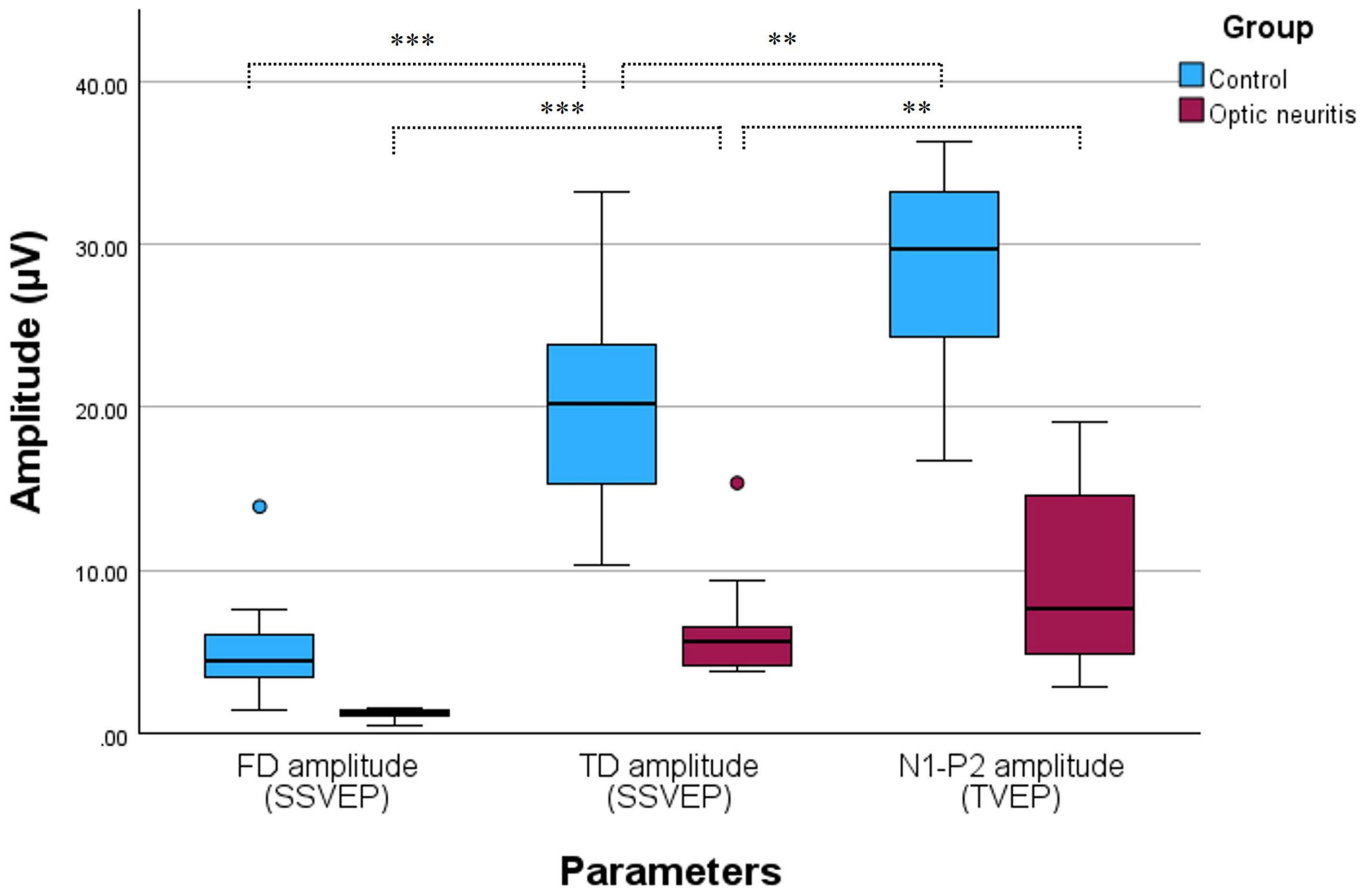
Box and whisker plot comparing SSVEP and TVEP amplitudes in the Control and Optic neuritis groups. The FD amplitude (blue: control, red: optic neuritis) was significantly smaller than the TD amplitude in both groups (control: *p* < 0.001; optic neuritis: *p* < 0.001). Additionally, the N1-P2 amplitude (TVEP) was significantly larger than the TD amplitude in both groups (control: *p* = 0.003; optic neuritis: *p* = 0.008). All statistical comparisons were performed with Bonferroni correction (adjusted significance threshold: *p* < 0.025). *p < 0.05, **p < 0.01, ***p < 0.001.

The FD and TD amplitude measured from SSVEP were compared to assess differences between frequency-domain and time-domain analyses. These comparisons are summarized in Figure 3, which presents a box and whisker plot of the FD, TD, and N1–P2 amplitudes across groups. In both the control and optic neuritis groups, the TD amplitude was significantly larger than the FD amplitude (control: *p* < 0.001; optic neuritis: *p* = 0.001).

To evaluate differences between steady-state and transient VEP, the TD amplitude from SSVEP was compared with the N1-P2 amplitude from TVEP. The N1-P2 amplitude was significantly larger than the TD amplitude in both the control group (*p* = 0.003) and the optic neuritis group (*p* = 0.008).

## 4 Discussion

This study compared the diagnostic potential of SSVEP and TVEP in detecting optic neuritis in dogs. Significant reductions in FD amplitude and SNR in the optic neuritis group underscore the utility of SSVEP in detecting optic nerve dysfunction through frequency-domain analysis.

Previous studies have supported the utility of SSVEP in identifying visual pathway dysfunctions. For instance, Nakanishi et al. reported reductions in SNR in multiple sclerosis patients, indicative of demyelination and axonal damage (23). Similarly, Zakaib et al. (24) demonstrated that SSVEP effectively detected visual pathway disruptions in children with optic pathway gliomas.

SSVEP isolates response amplitudes at specific stimulus frequencies, effectively minimizing broadband artifacts, such as muscle and retinal potentials, that can interfere with analysis (10). In contrast, TVEP waveforms are more susceptible to such artifacts, complicating peak identification for examiners (7, 17). Figure 2 illustrates this limitation, showing how significant noise interferes with TVEP, making peak differentiation challenging in a dog with optic neuritis.

Conversely, the SSVEP spectral response achieved an SNR greater than 3, surpassing the threshold for reliable analysis with a 0.3% risk of noise misclassification. These findings underscore the diagnostic advantages of SSVEP, particularly in noisy conditions where time-domain methods like TVEP are less effective (10, 17).

The GEE analysis further confirmed these results. Despite the significantly lower FD amplitude in the optic neuritis group (1.19 ± 0.26 μV, *p* < 0.001), the SNR remained above 3 (5.20 ± 3.50). This indicates that SSVEP can reliably validate response accuracy, even in cases of weak neural activity.

In this study, the N1-P2 amplitude of the TVEP was significantly lower in the optic neuritis group, while the control group had values around 25 μV, consistent with previous studies (3, 4, 22). Although the N1-P2 amplitude was significantly reduced, no delay in P2 latency was observed. This absence of latency prolongation may reflect axonal loss without demyelination, as previously reported in experimental models (21). However, TVEP, derived from time-domain waveforms, is inherently susceptible to waveform distortion and instability, which may obscure peak detection and introduce variability in latency measurements (25, 26). In addition, the limited sample size may have further reduced the ability to detect subtle latency changes. Therefore, both biological and methodological factors should be considered when interpreting this result.

This study did not compare the SNR of TVEP and SSVEP directly due to fundamental differences in calculation methods. In SSVEP, SNR is calculated in the frequency domain by isolating the stimulus frequency response and comparing it to surrounding noise (10). By contrast, TVEP SNR relies on time-domain methods, such as root mean square (RMS) or standard deviation of the signal. These methods lack standardization and do not provide a universally accepted way to assess data quality or noise levels in individual measurements (27).

To address this limitation, we employed an indirect approach. First, FD amplitude and TD amplitude were derived from the same SSVEP dataset to compare differences between frequency-domain and time-domain methods. Then, the TD amplitude from SSVEP was compared with the N1-P2 amplitude from TVEP to explore amplitude differences between transient and steady-state responses within the time domain.

The TD amplitude was significantly larger than the FD amplitude in both groups, likely due to the inclusion of artifact components in the time-domain analysis (28, 29). In contrast, the frequency-domain, by minimizing noise and artifact interference, may account for the smaller amplitude observed in this comparison (10).

When comparing the TD amplitude from SSVEP with the N1-P2 amplitude from TVEP, TVEP showed significantly larger amplitudes in both groups. This difference reflects the transient nature of TVEP, where each stimulus has sufficient time to fully elicit a distinct response, resulting in higher peak amplitudes (1). In contrast, the overlapping responses in SSVEP reduce apparent peak amplitudes (5). However, noise artifacts in TVEP may exaggerate peak amplitudes, potentially leading to overestimated values (26). Future studies should explore quantitative methods to better assess and minimize noise-related artifacts in TVEP.

Despite these limitations, SSVEP demonstrates clear advantages in frequency-domain analysis, with FD amplitude effectively distinguishing between control and optic neuritis groups. TVEP, while producing larger peak amplitudes, may suffer from overestimation due to noise artifacts. These findings emphasize the complementary strengths and limitations of each method and highlight the need for further research to standardize methodologies and explore their combined utility.

In addition to the analytical results, the practical feasibility and physiological implications of unsedated VEP recordings should be considered. To our knowledge, no studies have directly compared VEP recordings between sedated and conscious dogs, a previous report described a trend toward longer latencies and lower amplitudes in sedated animals compared to those under manual restraint, though these findings were not statistically analyzed (30). While flash VEP is generally recordable under sedation or anesthesia, deep anesthesia may suppress cortical responsiveness and reduce signal quality (2). Conversely, light sedation has been reported to suppress background electroencephalography (EEG) activity, potentially improving the SNR (30). These opposing effects highlight the complexity of sedation-related influences on VEP quality and should be taken into account when interpreting recordings obtained in awake animals.

Building on these physiological considerations, our experience also highlighted several practical aspects and technical challenges associated with unsedated VEP acquisition in conscious dogs. Among dogs that tolerated awake ERG, VEP acquisition was generally successful using a single handler supporting the mandible and neck.

However, the smaller amplitude of TVEP compared to ERG made the recordings more vulnerable to movement artifacts in some cases. For example, one dog exhibited excessive panting; while ERG was successfully recorded, the TVEP results were inconclusive due to motion-related noise interference, and this case was excluded from the analysis.

Unlike ERG, VEP measurement did not require repositioning of the electrode when recording from the contralateral eye, which allowed for a shorter session duration overall. As a result, bilateral VEP recordings were typically completed within 3–4 min of starting the procedure. In cases where fewer trials were obtained, TVEP was particularly susceptible to noise interference, and increasing the number of repetitions proved beneficial for reliable waveform analysis. Previous studies have commonly used 30 trials per eye; in our study, more than 50 trials were recorded per eye to enhance signal quality and improve waveform clarity (3, 4).

In this study, the reference electrode was placed at Fpz to maintain consistency with established canine VEP protocols (3, 4, 15, 30). While this site is commonly used, its proximity to the forehead and facial muscles may increase susceptibility to motion artifacts in awake animals. Among the three electrode sites used, Fpz caused the most discomfort or pain responses in the dogs during placement, and therefore the electrode at this site was always attached last. Cz was used as the ground electrode in accordance with standard practice and was therefore not considered as a reference site. Nonetheless, evaluating alternative reference locations that are less affected by movement or muscle activity may help improve signal quality in future studies involving conscious subjects.

This study is limited by its small sample size, which resulted from selecting seven cases and matching them with control dogs of similar breed and weight. While the breeds were not perfectly matched, efforts were made to include dogs with similar head sizes and body weights to minimize variability. The limited sample size may have reduced the statistical power to detect group differences, particularly for latency parameters such as TVEP P2 latency and SSVEP phase latency, where no statistically significant differences were observed. In such measures, the possibility of type II errors cannot be excluded (31). Therefore, these results should be interpreted with caution, and larger studies are warranted to validate the findings and improve their generalizability.

In addition, minor ocular conditions in some control animals, such as blepharitis and epiphora, may have affected VEP recordings by altering ocular surface stability or signal consistency. Although these conditions were clinically mild, subtle effects on signal quality cannot be entirely ruled out.

Another limitation is that the indirect comparison between SSVEP and TVEP limits the ability to directly assess their relative diagnostic capabilities. While receiver operating characteristic (ROC) curve analysis was considered, the small sample size could distort the results (32). Future research should incorporate ROC curve analysis to provide a more reliable evaluation of the diagnostic ability of both tests.

Finally, this study evaluated SSVEP at a single frequency of 8 Hz. However, human studies have explored multiple frequencies for SSVEP analysis (10). Future research should assess SSVEP at varying frequencies to understand its broader diagnostic applicability.

In conclusion, this pilot study provides preliminary evidence that frequency-domain SSVEP may offer a viable approach for detecting optic neuritis in dogs. The significantly reduced response amplitudes observed in affected animals support its utility in identifying visual pathway dysfunction. Additionally, consistent recordings without the need for sedation support its feasibility in clinical settings, particularly when sedation is contraindicated. Based on these findings, SSVEP may be considered for screening or follow-up evaluations in veterinary patients with optic nerve disorders. Further research with larger samples and varied stimulation parameters is necessary to validate its clinical utility and expand its applications.

## Data Availability

All data generated or analyzed during this study are included in this published article [and its supplementary information files]. The datasets used and/or analyzed during the current study are available from the corresponding author on reasonable request.
